# Divergent *in vivo* activity of non‐serotonergic and serotonergic VGluT3–neurones in the median raphe region

**DOI:** 10.1113/JP272036

**Published:** 2016-04-28

**Authors:** Andor Domonkos, Litsa Nikitidou Ledri, Tamás Laszlovszky, Csaba Cserép, Zsolt Borhegyi, Edit Papp, Gábor Nyiri, Tamás F. Freund, Viktor Varga

**Affiliations:** ^1^ Institute of Experimental MedicineHungarian Academy of SciencesBudapestHungary; ^2^ János Szentágothai Doctoral School of NeurosciencesSemmelweis UniversityBudapestHungary; ^3^ Present address: MTA‐ELTE‐NAP B‐Opto‐Neuropharmacology GroupEötvös Loránd UniversityBudapestHungary

## Abstract

**Key points:**

- The median raphe is a key subcortical modulatory centre involved in several brain functions, such as regulation of the sleep–wake cycle, emotions and memory storage.
- A large proportion of median raphe neurones are glutamatergic and implement a radically different mode of communication compared to serotonergic cells, although their *in vivo* activity is unknown.
- We provide the first description of the *in vivo*, brain state‐dependent firing properties of median raphe glutamatergic neurones identified by immunopositivity for the vesicular glutamate transporter type 3 (VGluT3) and serotonin (5‐HT). Glutamatergic populations (VGluT3+/5‐HT– and VGluT3+/5‐HT+) were compared with the purely serotonergic (VGluT3–/5‐HT+ and VGluT3–/5‐HT–) neurones.
- VGluT3+/5‐HT+ neurones fired similar to VGluT3–/5‐HT+ cells, whereas they significantly diverged from the VGluT3+/5‐HT– population. Activity of the latter subgroup resembled the spiking of VGluT3–/5‐HT– cells, except for their diverging response to sensory stimulation.
- The VGluT3+ population of the median raphe may broadcast rapidly varying signals on top of a state‐dependent, tonic modulation.

**Abstract:**

Subcortical modulation is crucial for information processing in the cerebral cortex. Besides the canonical neuromodulators, glutamate has recently been identified as a key cotransmitter of numerous monoaminergic projections. In the median raphe, a pure glutamatergic neurone population projecting to limbic areas was also discovered with a possibly novel, yet undetermined function. In the present study, we report the first functional description of the vesicular glutamate transporter type 3 (VGluT3)‐expressing median raphe neurones. Because there is no appropriate genetic marker for the separation of serotonergic (5‐HT+) and non‐serotonergic (5‐HT–) VGluT3+ neurones, we utilized immunohistochemistry after recording and juxtacellular labelling in anaesthetized rats. VGluT3+/5‐HT– neurones fired faster, more variably and were permanently activated during sensory stimulation, as opposed to the transient response of the slow firing VGluT3–/5‐HT+ subgroup. VGluT3+/5‐HT– cells were also more active during hippocampal theta. In addition, the VGluT3–/5‐HT– population, comprising putative GABAergic cells, resembled the firing of VGluT3+/5‐HT– neurones but without any significant reaction to the sensory stimulus. Interestingly, the VGluT3+/5‐HT+ group, spiking slower than the VGluT3+/5‐HT– population, exhibited a mixed response (i.e. the initial transient activation was followed by a sustained elevation of firing). Phase coupling to hippocampal and prefrontal slow oscillations was found in VGluT3+/5‐HT– neurones, also differentiating them from the VGluT3+/5‐HT+ subpopulation. Taken together, glutamatergic neurones in the median raphe may implement multiple, highly divergent forms of modulation in parallel: a slow, tonic mode interrupted by sensory‐evoked rapid transients, as well as a fast one capable of conveying complex patterns influenced by sensory inputs.

## Key points

- The median raphe is a key subcortical modulatory centre involved in several brain functions, such as regulation of the sleep–wake cycle, emotions and memory storage.
- A large proportion of median raphe neurones are glutamatergic and implement a radically different mode of communication compared to serotonergic cells, although their *in vivo* activity is unknown.
- We provide the first description of the *in vivo*, brain state‐dependent firing properties of median raphe glutamatergic neurones identified by immunopositivity for the vesicular glutamate transporter type 3 (VGluT3) and serotonin (5‐HT). Glutamatergic populations (VGluT3+/5‐HT– and VGluT3+/5‐HT+) were compared with the purely serotonergic (VGluT3–/5‐HT+ and VGluT3–/5‐HT–) neurones.
- VGluT3+/5‐HT+ neurones fired similar to VGluT3–/5‐HT+ cells, whereas they significantly diverged from the VGluT3+/5‐HT– population. Activity of the latter subgroup resembled the spiking of VGluT3–/5‐HT– cells, except for their diverging response to sensory stimulation.
- The VGluT3+ population of the median raphe may broadcast rapidly varying signals on top of a state‐dependent, tonic modulation.

**Abbreviations**
AP: anteroposterior
DV: dorsoventral
5‐HT: serotonin
5‐HT1AR: 5‐HT receptor type 1A
ISI: interspike interval
LFP: local field potential
ML: mediolateral
MRR: median raphe region
TpH2: tryptophan hydroxylase
VGluT3: vesicular glutamate transporter type 3

## Introduction

The ascending projection from the median raphe region (MRR; median raphe nucleus and paramedian raphe nucleus) is essential for a multitude of brain functions, such as the regulation of the sleep–wake cycle (Monti *et al*. 2008) or the modulation of oscillations (Jackson *et al*. 2008). The MRR consists of a heterogeneous collection of serotonergic, GABAergic and later identified glutamatergic neurones (Jacobs & Azmitia, 1992; Allers & Sharp, 2003; Jackson *et al*. 2009). Initial studies described the electrophysiological phenotype of serotonin (5‐HT)‐containing neurones, with a slow, regular firing pattern that changes with the stages of the sleep–wake cycle (Mosko & Jacobs, 1974). Nevertheless, a significant number of unidentified, fast spiking neurones, which were occasionally coupled to forebrain oscillations or behavioural phenomena, were also recorded (Sakai & Crochet, 2001; Viana DiPrisco *et al*. 2002). Mounting evidence suggested that the rapidly firing MRR neurones may belong to 5‐HT– and possibly GABAergic subgroups (Allers & Sharp, 2003; Calizo *et al*. 2011; Kirby *et al*. 2003). However, some fast firing MRR neurones with spikes coupled to hippocampal theta, the prominent limbic oscillation, were unexpectedly found to contain 5‐HT (Kocsis *et al*. 2006).

The discovery of the third isoform of the vesicular glutamate transporter (VGluT3) in 5‐HT+ neurones, amongst others, raised the possibility of a novel, uncharacterized form of modulation by co‐released glutamate (Fremeau *et al*. 2002; Schäfer *et al*. 2002). Glutamatergic synaptic signalling by monoaminergic cells has only recently been confirmed (Qi *et al*. 2014; Noh *et al*. 2010; Varga *et al*. 2009). Glutamate from MRR axons, in contrast to 5‐HT, selectively and powerfully recruits certain interneurone types in the hippocampus via synapses that contain both 5‐HT and VGluT3 or only VGluT3 (Varga *et al*. 2009). In parallel with the identification of fast glutamatergic modulation, a large fraction of MRR projection neurones was reported to be purely glutamatergic in addition to serotonergic and dual‐transmitter subpopulations (Hioki *et al*. 2010; Jackson *et al*. 2009; Szőnyi *et al*. 2014). However, the functional characteristics of VGluT3+ MRR neurones (i.e. whether they resemble the classic slow firing phenotype of 5‐HT+ neurones or belong to fast spiking subgroups or form a mixed population) remain to be determined. The relationship of their discharge pattern to forebrain activity states, as well as to oscillations, is unknown.

In the present study, we characterized the *in vivo* activity of VGluT3‐expressing (5‐HT+ and 5‐HT–) MRR neurones and compared them with VGluT3– subgroups (5‐HT+ and 5‐HT–). Because there is no appropriate genetic marker to undoubtedly separate these subgroups, we juxtacellularly recorded their activity followed by labelling and identification utilizing *post hoc* immunohistochemistry.

## Methods

### Animals

A total of 301 male Wistar rats, weighing 200–500 g, were used in the present study. All experiments were performed in accordance with the Institutional Ethical Codex, Hungarian Act of Animal Care and Experimentation (1998, XXVIII, section 243/1998) and the European Union guidelines (directive 2010/63/EU), and with the approval of the Institutional Animal Care and Use Committee of the Institute of Experimental Medicine of the Hungarian Academy of Sciences. All efforts were made to minimize pain and suffering and to reduce the number of animals used.

### Juxtacellular recording in the MRR

The rats were anaesthetized by i.p. injection of 20% urethane (Sigma‐Aldrich, St Louis, MO, USA; dose: 0.007 ml g^–1^ body weight). During the experiments, the temperature of animals was held constant by a homeothermic heating pad. A cranial window was drilled to access the MRR either from lateral [stereotactic co‐ordinates: anteroposterior (AP) +1.0 mm, mediolateral (ML) –1.4 to –2.0 mm from lambda and 15°angle] or from posterior (stereotactic co‐ordinates: AP –2.6 mm, ML 0 mm from lambda and 30°angle). For juxtacellular recording, filamented borosilicate micropipettes were filled with 0.5 M NaCl containing 2% Neurobiotin (Vector Laboratories, Inc., Burlingame, CA, USA; impedance of micropipettes: 20–45 MΩ). After reaching the target zone (6900–8500 μm from brain surface), the recording pipette was slowly (0.8–1 μm s^–1^) advanced using a micropositioner (EXFO, Quebec, Canada). The juxtacellular MRR unit signal was filtered between 0.1 and 5 kHz and amplified (gain: 500 or 1000) using an AxoClamp 2B amplifier (Axon Instruments, Foster City, CA, USA) and a LinearAmp signal conditioner (Supertech, Pécs, Hungary).

### Electrode implantation into the hippocampus and prefrontal cortex

Additional holes were drilled for implantation of monopolar stainless steel wires into dorsal CA1 of the hippocampus [AP –4.5 mm, ML –2.5 mm or +2.5 mm from bregma and 0°angle, dorsoventral (DV): 2300–2400 μm from brain surface] and occasionally into the infralimbic part of prefrontal cortex (AP +2.7 mm, ML +0.5 to +0.6 mm from bregma and 0°angle, DV: 4500 μm from brain surface) to record local field potentials (LFP) filtered between 0.3 and 2 kHz (gain: 2000 or 5000), using an AC‐coupled amplifier (BioAmp; Supertech). A silver wire served as a common ground was placed below the neck skin. All electrophysiological data were digitized at 10 kHz using a Micro1401 mkII or a Micro3 1401 (Cambridge Electronic Design, Cambridge, UK) controlled by Spike2 (Cambridge Electronic Design).

### Recording protocol

Spontaneous and sensory stimulation‐evoked activity of MRR neurones simultaneous with hippocampal and occasionally prefrontal cortical LFP was registered. Tail pinch served as the sensory stimulation. The recording duration of spontaneous activity was 2–10 min (except for one case in which 1 min spontaneous activity was saved). In all experiments, the tail pinch was applied by the same small crocodile clip (the serrated surface was covered by thin plastic tape) for 30 s. In five cases, the tail pinch duration was 60 s. If the first stimulation had not evoked brain state change after at least 1 min, a second tail pinch was applied using a stronger clip. Only the first effective tail pinch was considered when the data were analysed. In the case of one neurone, no tail pinch was executed, and therefore the data from this recording were used only in spike half‐width analysis and phase preference calculation.

### Labelling of recorded neurones

In 166 animals (from a total of 301 rats), successful recordings were followed by Neurobiotin‐labelling of the neurones: 1–10 nA current (gradually increased until current‐modulated neuronal firing was detected) was applied via the recording pipette, in parallel with a cathodal DC current of 0.5–0.9 nA amplitude. The duration of labelling was 2–10 min (Pinault 1996).

### Histological processing

After successful labelling, animals were transcardially perfused with saline followed by Zamboni's fixative or 4% paraformaldehyde (survival time after labelling varied between 10 and 150 min). Brains were cut to 40–80 μm thick coronal slices on a vibrotome (Leica Microsystems, Wetzlar, Germany). After extensive washing in 0.1 m phosphate buffer (pH 7.4), the sections were transferred into Tris‐buffered saline (Sigma‐Aldrich) (pH 7.4). Subsequent to this step, all the washes and serum dilutions were performed in Tris‐buffered saline. Neurobiotin‐filled neurones were identified by Alexa488‐conjugated Streptavidin staining (Life Technologies, Grand Island, NY, USA, catalogue no.: S‐32354, dilution 1:2000), neurochemical content (5‐HT and VGluT3) was screened by immunofluorescence [primaries: rabbit anti‐5‐HT (ImmunoStar Inc., Hudson, WI, USA, catalogue no.: 20080, dilution 1:10000) and guinea pig anti‐VGluT3 (Merck, Darmstadt, Germany, catalogue no.: AB5421, dilution 1:2000); secondaries: Dylight405‐conjugated donkey anti‐rabbit (Jackson ImmunoResearch, West Grove, PA, USA, catalogue no.: 711‐475‐152, dilution 1:400) and Cy3‐conjugated donkey anti‐guinea‐pig (Jackson Immunoresearch, catalogue no.: 706‐166‐148, dilution 1:200)]. Fluorescence signals were inspected using an Axioplan 2 microscope (Carl Zeiss, Oberkochen, Germany) with a DP70 CCD‐camera (Olympus, Tokyo, Japan) or using an A1R confocal laser scanning microscope (Nikon, Tokyo, Japan).

### Reconstruction of the cells and the recording positions

Neurobiotin signal was intensified using Nickel‐DAB (2,3‐diaminobenzidine; Sigma‐Aldrich) chromogen developed by avidin‐biotinylated horseradish peroxidase complex (Elite ABC; Vector Laboratories, Inc., Burlingame, CA, USA). Dendritic arborization of neurones with good labelling quality was three‐dimensionally reconstructed using Neurolucida software (MBF Bioscience, Williston, VT, USA) and an Axioplan 2 microscope. The maximal diameter of the coronal projection of dendritic arborization was measured. The position of recovered neurones, as well as the position of LFP recording wires (except in some experiments; see Fig. 6 *E*) was determined based on a stereotaxic atlas of the rat brain (Paxinos & Watson, 2005).

### Selection criteria of cells

**Figure 1:**
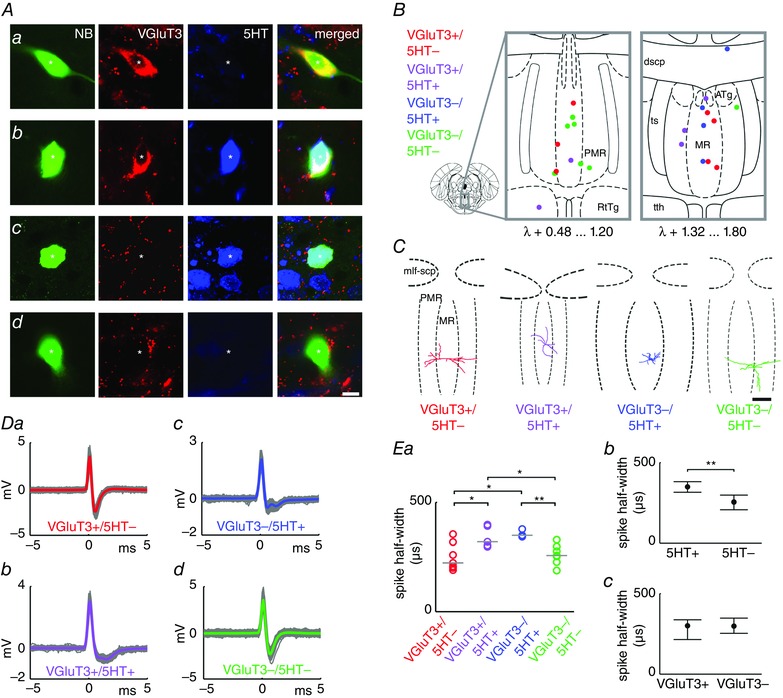
**Examples and recording positions of all neurochemically identified MRR neurones** Immunofluorescence images of example Neurobiotin (NB)‐filled neurones: VGluT3+/5‐HT– (*Aa*), VGluT3+/5‐HT+ (*Ab*), VGluT3–/5‐HT+ (*Ac*) and VGluT3–/5‐HT– (*Ad*) neurones. Asterisks indicate the soma. Scale bar = 10 μm. *B*, coloured dots indicate the position of recorded and neurochemically identified neurones overlaid onto two representative coronal diagrams of MRR (MR + PMR, based on Paxinos & Watson, 2005). ATg, anterior tegmental nucleus; dscp, decussation of the superior cerebellar peduncle; MR, median raphe nucleus; PMR, paramedian raphe nucleus; RtTg, reticulotegmental nucleus of the pons; ts, tectospinal tract; tth, trigeminothalamic tract. *C*, coronal projections of three‐dimensionally reconstructed dendritic arborization of four representative neurones. mlf‐scp, medial longitudinal fascicle and superior cerebellar peduncle; MR, median raphe nucleus; PMR, paramedian raphe nucleus. Scale bar = 300 μm. *Da*–*Dd*, spike waveform averages (plotted in colour) of corresponding neurones in (*A*). *Ea*, spike half‐width (grey lines: group medians). Kruskal–Wallis ANOVA: *P* = 0.0146. Wilcoxon rank sum test: *P < 0.05; **P < 0.01. Data grouped on the basis of 5‐HT (*Eb*) and VGluT3 (*Ec*) content (median + interquartile range).

In 78 out of 166 experiments, the labelled neurones were undoubtedly recovered. In some of these animals, two neurones were recorded and identified. In a few experiments, either the recording position and the location of the Neurobiotin‐filled neurone did not match perfectly or multiple somata were recovered close to each other. For all of these cases, the recordings were excluded from further analysis. By applying these restrictions, we recovered 60 MRR neurones. From that pool, only cells with unequivocal identification by immunofluorescence were involved in the present study, resulting in a total of 23 neurones (Fig. 1 *B*).

### Data analysis

Spikes in unit recordings were detected using Spike2 software, controlled by visual inspection to remove artefacts, and time stamps corresponding to the spike peaks were exported to the MATLAB (MathWorks Inc., Natick, MA, USA) environment, where custom‐written routines and scripts were run for further analysis. To calculate the spike waveform average, data in ±5 ms time windows around spike peaks were interpolated linearly to obtain a waveform oversampled at 1 MHz. Spike half‐width was determined as the duration of the action potential halfway between the baseline and the peak of the waveform average. For calculation of spike averages, all data from pre‐labelling recordings (i.e. including spontaneous and sensory stimulation periods) were used.

### Sensory stimulation‐evoked response

The sensory stimulation‐evoked response was tested in the first 30 s of tail pinch periods. Transient change was defined as the ratio of the normalized firing rate in 0–1 s *vs*. 0–5 s after the start of stimulation. Permanent change was defined as the difference of the normalized firing rate 0–15 s before and 1–16 s after the start of stimulation.

### Firing rate and interspike interval (ISI) distribution

The firing rate (event count / time in seconds) and the coefficient of variation of ISIs (SD/mean of ISI) were calculated for both spontaneous firing and sensory stimulation. For visualization purposes, we generated ISI distribution plots with the same logarithmically increasing bin range for all neurones. This bin range was delimited by the shortest and the longest ISI of all the recordings. Because the widely used parameter, the coefficient of variation, was reported as an underestimate of firing variability in some cases (Ditlevsen & Lansky, 2011), we calculated the Shannon‐entropy of ISI distribution as well (Huang *et al*. 2014). For calculation of the Shannon‐entropy of ISI distribution (absolute value of summed products of ISI distribution probabilities along all non‐zero bins and their respective second base logarithm), we used the Freedman–Diaconis rule to assess the optimal bin width individually for each neurone (spontaneous firing and sensory stimulation were also separated). The Freedman–Diaconis rule is an algorithm for optimal bin size estimation of data with heavy‐tailed distribution (Freedman & Diaconis, 1981). If there was no spiking during a condition, the coefficient of variation and Shannon‐entropy of ISI distribution resulted in a zero value.

### Rhythmic firing

The firing rhythmicity was evaluated by autocorrelograms of action potentials (spontaneous and sensory stimulated conditions were separated). Event probability was calculated with ±2 s lag around spike peaks in 50 ms time bins. In cases where rhythmic modulation was detected, the modulation frequency was calculated from the interval between peaks of autocorrelogram side lobes (not counting the central lobe).

### State‐dependent firing

Continuous wavelet transform of the hippocampal LFP was used to determine the hippocampal state based on the frequency of oscillation. To generate the continuous wavelet transform, the LFP raw signal was down‐sampled to 400 Hz, 50 Hz noise was filtered out (2048‐order band stop finite impulse response filter with 49–51 Hz cut‐off designed by MATLAB built‐in ‘fir1’ function), and then the normalized signal (the mean was subtracted and then divided by the SD) was decomposed by a Morlet kernel function with exponential scaling (Torrence & Compo, 1998). The wavelet power spectrum (i.e. the squared absolute value of the wavelet transform coefficient) was divided by the scale vector for bias rectification (Liu *et al*. 2007). Finally, scales below 0.9 Hz (possibly corresponding to any low‐frequency artefacts introduced at the onset or offset of the tail pinch) were cut and not used in further analysis. Theta and non‐theta states of the spontaneous, non‐stimulated condition were differentiated. Theta was detected when the summed power of the wavelet power spectrum in the band at 2.5–6.0 Hz was higher than in the band below 2.5 Hz; otherwise, the hippocampal state was identified as non‐theta. Spikes of MRR cells were counted during the theta and non‐theta states (in ±1.25 ms time windows because of 400 Hz sampling rate) and divided by the duration of the given state to obtain the firing frequency. The theta and non‐theta states were differentiated only during spontaneous activity; the sensory stimulation was not included in this analysis. The ISIs during theta *vs*. non‐theta states were separated, although the ISIs overlapping with state transitions were excluded. In addition, the coefficient of variation and the Shannon‐entropy of distribution of these ISI groups were calculated.

### Phase preference

The frequency of the dominant oscillation was determined as the frequency with highest spectral power in non‐bias‐rectified wavelet transform. The instantaneous phase values coinciding with spikes were extracted from wavelet coefficients. Events concurring during theta (dominant frequency of LFP: 2.5–6.0 Hz) and non‐theta (slow; dominant frequency of LFP: 0.9–2.5 Hz) oscillations were segregated. The phase values according to theta and non‐theta groups were also merged. Spontaneous and stimulated conditions were also separated. The uniformity of spike‐coupled instantaneous phase distribution (i. e. phase preference) was tested by Watson's test and, in cases where the distribution was found to deviate from uniformity, the mean phase and mean vector length were determined. To generate phase histograms, the circle was divided into 20° bins. Neurones must have had at least 50 spikes during a given condition (spontaneous or stimulated) and 40 spikes during spontaneous theta and non‐theta oscillations to be included in the analysis.

### Statistical analysis

Individual values and medians are presented. In some cases, medians and interquartile range are plotted. Group differences were tested by Kruskal–Wallis ANOVA and the two‐sided Wilcoxon rank sum test. Divergence between theta and non‐theta, as well as between spontaneous and stimulated conditions, was assessed using a sign test. In the case of maximal dendritic diameter analysis, data are only presented in the text.

## Results

Spontaneous activity of the MRR neurones was juxtacellularly recorded simultaneously with LFP in the hippocampus, the major limbic target, and occasionally in the prefrontal cortex, the main cortical input of the MRR in urethane‐anaesthetized male Wistar rats. Tail pinch as sensory stimulation was applied for 30 s after the spontaneous activity recording.

Unequivocal neurochemical identification (see selection criteria in Methods) was possible in 23 neurones out of 60 recovered MRR cells, which resulted in seven VGluT3+/5‐HT–, five VGluT3+/5‐HT+, four VGluT3–/5‐HT+ and seven VGluT3–/5‐HT– neurones being included in the present study (Fig. 1 *A*). Two out of the nine 5‐HT+ neurones were found in transition zones outside the MRR (Fig. 1 *B*), although these cells were no different in any respect from MRR 5‐HT+ neurones, and so all the further analyses contain the data from these cells. The dendritic arborization of 15 neurones was three‐dimensionally reconstructed (Fig. 1 *C*): the dendritic tree of VGluT3+/5‐HT– neurones span a larger distance as a result of long, relatively branchless dendrites (group median diameter of dendritic arbor: 752.9 μm, interquartile range: 594.9–859.9 μm) compared to the two 5‐HT+ (VGluT3+ and VGluT3–, group medians: 536.2 and 510.1 μm, interquartile ranges: 488.0–713.8 μm and 325.5–694.6 μm, respectively) and the VGluT3–/5‐HT– (group median: 522.1 μm, interquartile range: 272.6–841.5 μm) populations (number of reconstructed cells: VGluT3+/5‐HT–, *n* = 5; VGluT3+/5‐HT+, *n* = 4; VGluT3–/5‐HT+, *n* = 2; VGluT3–/5‐HT–, *n* = 4). However, based on these data, there was no significant morphological difference between the MRR subpopulations (Kruskal–Wallis ANOVA: *P* = 0.6481). Comparison of the action potential half‐width from both spontaneous and stimulated conditions revealed that the 5‐HT– population had narrower spikes (group median of VGluT3+/5‐HT–: 223.5 μs and VGluT3–/5‐HT–: 257.1 μs) compared to their 5‐HT+ counterparts (group median of VGluT3–/5‐HT+: 319.9 μs and VGluT3+/5‐HT+: 349.3 μs) (Fig. 1 *D* and *E)*.

### Firing rate and firing variability during spontaneous activity

**Figure 2:**
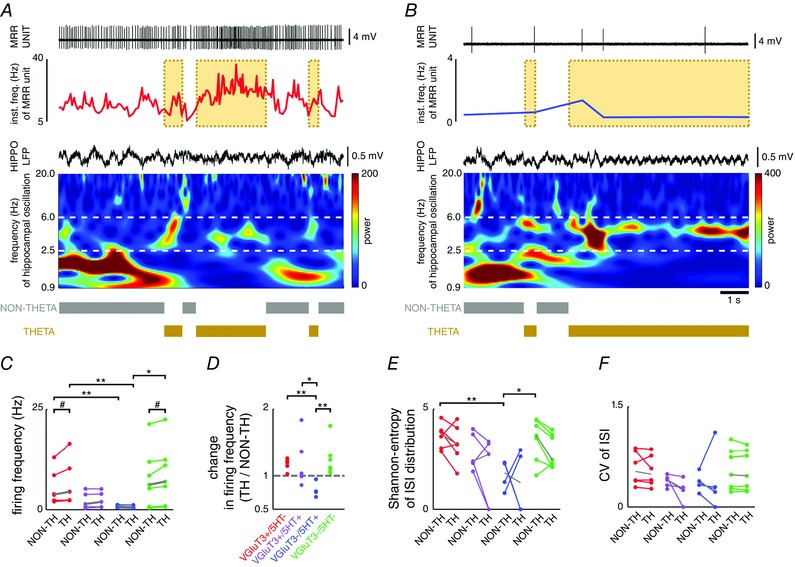
**State‐dependent firing of MRR neurones** Firing of a VGluT3+/5‐HT– (*A*) and a VGluT3–/5‐HT+ (*B*) neurone in relation to hippocampal theta and non‐theta states. Upper: raw unit recording (high‐pass filtered at 100 Hz) and instantaneous firing frequency. Bottom: simultaneous wide band (0.3–5000 Hz) hippocampal LFP, spectral power of wavelet decomposition of LFP and definition of theta and non‐theta states. Note the elevated baseline activity of the VGluT3‐positive neurone marked by the yellow background area during a hippocampal theta episode. *C*, firing frequency during non‐theta and theta states. Group medians are plotted in grey. Kruskal–Wallis ANOVA: non‐theta: *P* = 0.0769; theta: *P* = 0.0168. Two‐sided Wilcoxon rank sum test: *P < 0.05, **P < 0.01. Sign test: ^#^ *P* < 0.05. *D*, ratio of theta *vs*. non‐theta firing frequency. Kruskal–Wallis ANOVA: *P* = 0.0264. Two‐sided Wilcoxon rank sum test: *P < 0.05; **P < 0.01. *E*, Shannon‐entropy of ISI distribution. Group medians are plotted in grey. Kruskal–Wallis ANOVA: non‐theta: *P* = 0.0365; theta: *P* = 0.2603. Two‐sided Wilcoxon rank sum test: *P < 0.05, **P < 0.01. *F*, coefficient of variation of ISI. Group medians are plotted in grey. Kruskal–Wallis ANOVA: non‐theta: *P* = 0.5563; theta: *P* = 0.3216.

Spontaneous recording segments were divided into non‐theta and theta epochs, which enabled the analysis of state‐dependent activity (Fig. 2 *A* and *B*). VGluT3+/5‐HT– cells fired significantly faster than VGluT3–/5‐HT+ cells (Fig. 2 *C*) during both non‐theta (group median firing rate: 3.78 and 0.82 Hz, respectively) and theta states (group median firing rate: 4.48 and 0.58 Hz, respectively). VGluT3+/5‐HT+ neurones not diverging consequentially from the previous populations, represented a transition subgroup between them (group median firing rate during non‐theta: 1.43 Hz and during theta: 1.95 Hz) (Fig. 2 *C*). VGluT3–/5‐HT– neurones (at group level) fired fast (group median firing rate during non‐theta: 6.23 Hz and during theta: 7.00 Hz) similar to VGluT3+ neurones (Fig. 2 *C*). Spontaneous transition from non‐theta to theta was accompanied by the activation of VGluT3+/5‐HT– and VGluT3–/5‐HT– neurones (Fig. 2 *A, C* and *D*). By contrast, the firing rate of VGluT3–/5‐HT+ neurones was lower during theta, setting them apart from even VGluT3+/5‐HT+ neurones that did not show a group‐level state‐preference (Fig. 2 *B–D*). Thus, the firing rate ratio between non‐theta and theta epochs revealed a significant activation of VGluT3+/5‐HT– and VGluT3–/5‐HT– during theta. An opposite direction of change was found in the case of VGluT3–/5‐HT+ neurones, whereas both facilitation and suppression was detected in the VGluT3+/5‐HT+ population (Fig. 2 *D*). The firing variability of VGluT3+/5‐HT– and VGluT3–/5‐HT+ neurones during non‐theta state was different, as denoted by Shannon‐entropy of ISI distribution, although, during theta, there was no significant variability‐difference across neurochemical groups (Fig. 2 *E*). The coefficient of variation of ISI showed no consequent distinction either during non‐theta or theta states (Fig. 2 *F*).

**Figure 3:**
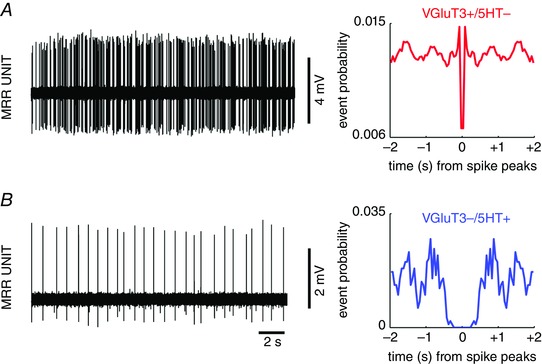
**Two MRR neurones exhibited spontaneous rhythmic firing** Selected recording trace (left) and autocorrelogram (right) of VGluT3+/5‐HT– (*A*) and VGluT3–/5‐HT+ (*B*) neurones.

According to recent studies, rhythmic firing was reported in a subgroup of both 5‐HT+ and 5‐HT– neurones (Allers & Sharp, 2003; Kocsis *et al*. 2006). Therefore, we assessed the rhythmic modulation of our sample of identified neurones. Based on the analysis of autocorrelograms, a rhythmic component in the spike train of only one VGluT3+/5–HT– and one VGluT3–/5‐HT+ neurone was detected. The former reflected the low frequency (1.33 Hz), periodic fluctuation of spike density, whereas the latter corresponded to regular single spiking (Fig. 3).

### Effect of sensory stimulation

**Figure 4:**
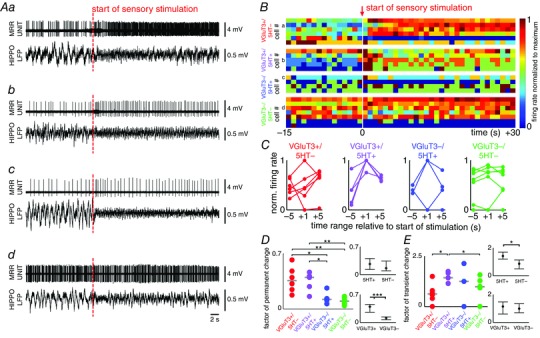
**Divergent effect of sensory stimulation on the activity of MRR neurones** Raw recordings of VGluT3+/5‐HT– (*Aa*), VGluT3+/5‐HT+ (*Ab*), VGluT3–/5‐HT+ (*Ac*) and VGluT3–/5‐HT– (*Ad*) neurones (red dashed line: start of 30 s long tail pinch). Upper: high‐pass filtered (at 100 Hz) MRR unit recording. Bottom: wide band (0.3–5000 Hz) hippocampal LFP. *B*, response of all identified neurones to sensory stimulation (white line: start of 30 s long tail pinch). Examples presented in (*A*) are indicated on the left. Firing rate was calculated in 1 s time bins and normalized to the maximum. Cells in each neurochemical category were ordered by the highest firing rate within the extracted recording segment. *C*, transient firing rate change after sensory stimulation. Normalized firing in given time ranges were condensed from the data shown in (*B*). *D* and *E*, quantification of permanent (*D*) and transient (*E*) firing rate changes. Kruskal–Wallis ANOVA: transient changes: 0.1065; permanent changes: 0.0028. Wilcoxon rank sum test: *P < 0.05; **P < 0.01; ***P < 0.001. Right: data are grouped on the basis of 5‐HT (upper) and VGluT3 (bottom) content (median + interquartile range).

The activity of MRR neurones was affected in a substantially divergent manner by sensory stimulation (Fig. 4 *A* and *B*). The firing rate of VGluT3+ cells (both 5‐HT+ and 5‐HT–) changed permanently, which was manifested as an elevation in the majority (eight out of 11) or cessation of firing in case of one VGluT3+ cell (Fig. 4 *C* and *D*). By contrast, most of the VGluT3–/5‐HT+ neurones only transiently changed their firing at stimulus onset (three increased and one stopped spiking) (Fig. 4 *C* and *E*). Notably, VGluT3+/5‐HT+ cells responded by both transient, short lasting (five out of five) and sustained (four out of five) acceleration. VGluT3–/5‐HT– neurones did not change their firing rate in response to sensory stimulation.

**Figure 5:**
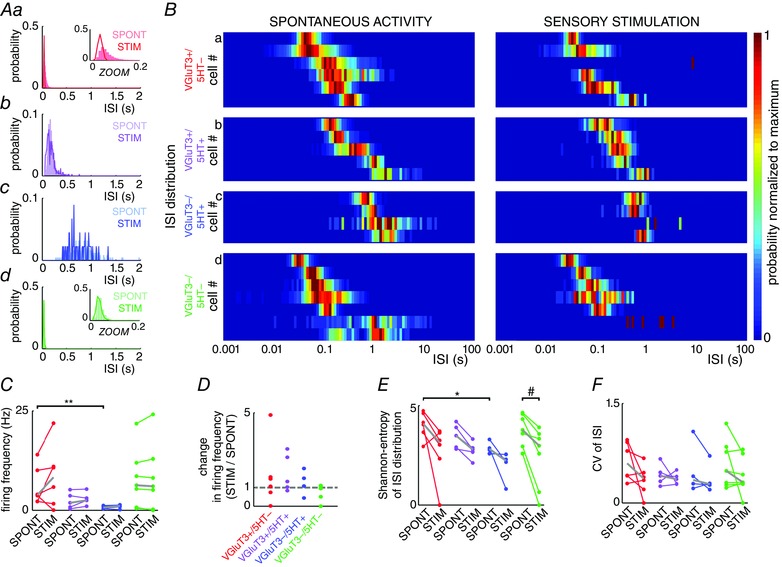
**Firing frequency and ISI distribution of MRR neurones during spontaneous activity and sensory stimulation** ISI distribution of example neurones: VGluT3+/5‐HT– (*Aa*), VGluT3+/5‐HT+ (*Ab*), VGluT3–/5‐HT+ (*Ac*) and VGluT3–/5‐HT– (*Ad*) neurones. Data from spontaneous activity and sensory stimulated conditions are separated. *B*, ISI distribution of all identified neurones (excluding the single VGluT3+/5‐HT– neurone without sensory stimulation), normalized to the maximum of the given neurone. Examples shown in (*A*) are indicated on the left side. Cells are ordered by the peaks closest to the shortest ISI. Note that the *x*‐axis has logarithmic scaling. *C*, firing frequency during spontaneous and stimulated conditions. Group medians are plotted in grey. Kruskal–Wallis ANOVA: spontaneous activity: *P* = 0.0424; stimulation: *P* = 0.1992. Two‐sided Wilcoxon rank sum test: **P < 0.01. *D*, ratio of stimulation evoked *vs*. spontaneous firing frequency. Kruskal–Wallis ANOVA: *P* = 0.3191. *E*, Shannon‐entropy of ISI distribution. Group medians are plotted in grey. Kruskal–Wallis ANOVA: spontaneous activity: *P* = 0.0605; stimulation: *P* = 0.3679. Two‐sided Wilcoxon rank sum test: *P < 0.05. Sign test: ^#^ *P* < 0.05. *F*, coefficient of variation of ISI. Group medians are plotted in grey. Kruskal–Wallis ANOVA: spontaneous activity: *P* = 0.5789; stimulation: *P* = 0.8647.

Comparing the ISI distributions during spontaneous (without separation of non‐theta and theta) and stimulation‐evoked activity uncovered only subtle alteration of shape, indicating the lack of a firing pattern switch (Fig. 5 *A* and *B*). Although the VGluT3+/5‐HT– population fired faster than the VGluT3–/5‐HT+ cells even during stimulation (Fig. 5 *C*), as in the spontaneous condition (both non‐theta and theta epochs) (Fig. 2 *C*), stimulation altered the activity of individual neurones in significantly divergent directions compared to the spontaneous non‐theta to theta transition (Fig. 2 *D vs*. Fig. 5 *D*). Importantly, during stimulus delivery, ISI variability was reduced relative to pre‐stimulation periods throughout all neurochemical subgroups, as reflected by both the narrowing of ISI distributions (Fig. 5 *B*) and the drop of entropy (Fig. 5 *E* and *F*). Rhythmic modulation of firing was not detectable during sensory stimulation in any cells.

### Phase coupling to forebrain oscillations

**Figure 6:**
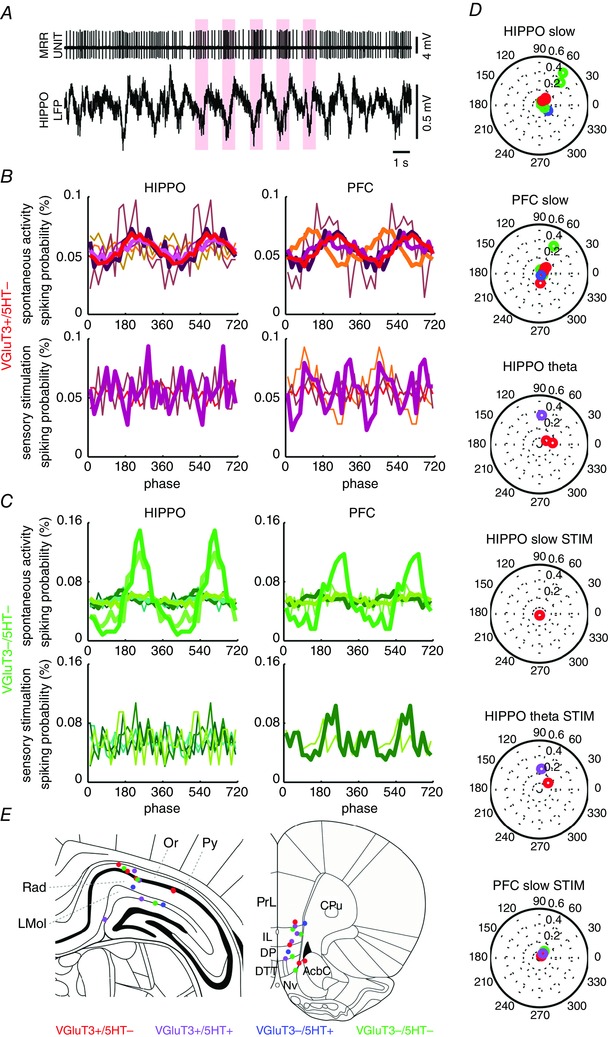
**Phase‐coupling of MRR neurones to forebrain oscillations** *A*, raw recording of a VGluT3+/5‐HT– neurone coupled to troughs of hippocampal slow oscillation. Upper: high‐pass filtered (at 100 Hz) MRR unit recording. Bottom: wide band (0.3–5000 Hz) hippocampal LFP. Slow waves with coupled spikes are highlighted. *B* and *C*, hippocampal (left) and prefrontal (right) slow oscillation phase preference. VGluT3+/5‐HT– (*B*) and VGluT3–/5‐HT– cells (*C*) were typically locked to hippocampal and/or prefrontal slow (0.9–2.5 Hz) oscillations. All cells are plotted in different colours, enabling the comparison of hippocampal and prefrontal preferences. Thick lines: significant difference from uniform distribution (*P* < 0.05, Watson's test). *D*, polar histograms show the mean vector length (radius, bold numbers) at the preferred phase (angle in degrees) during conditions when at least one neurone had a significant phase preference to slow or theta band oscillations. Colour codes neurochemical content. *E*, LFP recording positions in hippocampus (left: bregma –4.56 mm) and prefrontal cortex (right: bregma + 2.76 mm). Colour codes the neurochemical content of the MRR neurone recorded in the given experiment. Note that, in the case of some experiments (hippocampal recording site: *n* = 2 VGluT3+/5‐HT–, *n* = 1 VGluT3+/5‐HT+, *n* = 1 VGluT3–/5‐HT+, *n* = 4 VGluT3–/5‐HT–; prefrontal recording site: *n* = 3 VGluT3+/5‐HT–, *n* = 1 VGluT3+/5‐HT+, *n* = 1 VGluT3–/5‐HT+, *n* = 3 VGluT3–/5‐HT–), the recording position was not recovered. Only the structures close to recording area are indicated based on Paxinos & Watson, 2005). Hippocampus: LMol, lacunosum moleculare layer of the hippocampus; Or, oriens layer of the hippocampus; Py, pyramidal cell layer of the hippocampus; Rad, radiatum layer of the hippocampus. Prefrontal cortex: AcbC, accumbens nucleus, core; CPu, caudate putamen (striatum); DP, dorsal peduncular cortex; DTT, dorsal tenia tecta; IL, infralimbic cortex; Nv, navicular nucleus of the basal forebrain; PrL, prelimbic cortex.

The temporal relationship between the firing of MRR neurones and the phase of hippocampal and prefrontal oscillations was analysed. 5‐HT– (both VGluT3+ and VGluT3–) cells were weakly phase‐coupled to hippocampal and/or prefrontal slow oscillation, which disappeared during sensory stimulation (Fig. 6 *A–C*). Although 5‐HT– neurones were characteristically modulated by forebrain slow oscillation, the mean vector length corresponding to the strength of phase coupling was low in most cases (Fig. 6 *D*). Phase coupling to hippocampal theta was observed for one VGluT3+/5‐HT+ neurone (Fig. 6 *D*). VGluT3–/5‐HT+ cells showed weak coupling to hippocampal or prefrontal activity.

## Discussion

In the present study, we provide the first description of the *in vivo*, brain state‐dependent firing properties of the previously uncharacterized glutamatergic neurone populations in the MRR. We demonstrate that VGluT3+/5‐HT– neurones fundamentally diverged from VGluT3+/5‐HT+ cells in several aspects. VGluT3+/5‐HT– neurones, resembling VGluT3–/5‐HT– cells, had narrow action potentials and fired faster and more variably compared to their VGluT3+/5‐HT+ counterparts, whereas the latter population emitted broad action potentials at lower frequency in a regular fashion and, as such, overlapped with the VGluT3–/5‐HT+ subgroup. The activity of 5‐HT– populations was characteristically higher during hippocampal theta compared to non‐theta state, as opposed to the lack of group‐level state‐preference in the 5‐HT‐containing subgroups. In the case of some VGluT3+/5‐HT– and VGluT3+/5‐HT+ cells, weak modulation by forebrain oscillations was also detected. The response to sensory stimulation differentiated all four neurochemical categories: VGluT3+/5‐HT– neurones exhibited elevated activity throughout stimulus delivery, whereas VGluT3–/5‐HT+ cells were activated only at stimulus onset. VGluT3+/5‐HT+ neurones were characterized by the combined response of a transient acceleration and subsequent long‐lasting increase of activity. VGluT3–/5‐HT– cells were not significantly affected by the sensory stimulus.

### Technical considerations

The currently available, MRR‐related recombinase expressing mouse strains, including VGluT3‐Cre (Tatti *et al*. 2014), ePet‐Cre (Scott *et al*. 2005), Pet1_210_‐Cre (Pelosi *et al*. 2014), TpH2‐Cre (Weber *et al*. 2009, 2011) or SERT‐Cre (Zhuang *et al*. 2005) animals, cannot unequivocally differentiate the most prominent MRR subpopulations. VGluT3 is also expressed by 5‐HT+ cells, but, as demonstrated in the present study, they are fundamentally different from the VGluT3+/5‐HT– group, rendering presently known serotonergic and glutamatergic markers unusable for tagging only the 5‐HT– glutamatergic population. Therefore, the only feasible technique is labelling after single‐cell recording and *post hoc* neurochemical identification (Pinault 1996). This constraint led us to deploy juxtacellular recording under urethane‐anaesthesia, which reportedly maintains the brain close to natural sleep conditions (Clement *et al*. 2008; Pagliardini *et al*. 2013) and provides a mechanically stable preparation, and, as such, enables both the lengthening of recordings and an increase in the yield of labelling attempts. Moreover, the spontaneous alternation of brain states characteristic to urethane‐anaesthesia was pivotal for investigating the state‐dependent activity of MRR neurones.

### Questions about the identity of the identified populations

First, our findings demonstrate that, solely on the basis of electrophysiological criteria, the major neurone classes in the MRR cannot be separated. Even the well‐established pharmacological methods relying on the inhibitory effect of 5‐HT receptor type 1A (5‐HT1AR) agonists on serotonergic activity (Viana Di Prisco *et al*. 2002) are insufficient because of the overlap between the VGluT3+ and 5‐HT+ subgroups (as reported in the present study), the lack of 5‐HT1AR in the case of some 5‐HT+ raphe neurones (Kiyasova *et al*. 2013) and the indirect disfacilitation of 5‐HT– cells by dampened 5‐HT levels (Liu *et al*. 2000). A striking observation of the present study is the overlap between VGluT3+/5‐HT– and VGluT3–/5‐HT–, putative GABAergic fast firing neurones, implying that a significant proportion of rapidly spiking cells reported earlier in blind recording studies (Kocsis & Vertes, 1996; Viana Di Prisco *et al*. 2002; Wang *et al*. 2015) may have belonged to the VGluT3+/5‐HT– subclass.

The intermediate phenotype of VGluT3+/5‐HT+ neurones raises two radically different possibilities. First, the ‘pure’ glutamatergic and serotonergic populations correspond to the two extrema of a spectrum with the VGluT3+/5–HT+ cells as a transitional form inbetween. As documented for several other neurone types (Baudry *et al*. 2010; Dulcis *et al*. 2013; Dehorter *et al*. 2015), these populations may transform into each other by yet unknown mechanisms. The negative correlation of VGluT3 and tryptophan hydroxylase (TpH2) expression (Okaty *et al*. 2015) may also support this possibility: all non‐GABAergic raphe neurones probably express certain (but, in several cases, immunohistochemically undetectable) amounts of VGluT3 and TpH2. Alternatively, VGluT3+/5‐HT– MRR neurones may form a ‘truly’ discrete class, without the possibility of conversion into 5‐HT+ cells, which is supported by their substantially different firing behaviour. Nevertheless, either of these alternatives will have a profound impact on our understanding of the operation of the serotonergic system.

### Basic electrophysiological characteristics of MRR neurones: spike width, frequency and variability

Spike width, firing frequency and ISI variability have been used as basic electrophysiological characteristics to separate various neurone types, including 5‐HT+ and 5‐HT– cells. These parameters clearly segregated the VGluT3+ population in correlation with 5‐HT content. The 5‐HT– subgroups fired narrow action potentials at high frequency in a more irregular manner compared to the slow, regular firing of broad spikes by the 5‐HT+ subgroup. In our sample, the VGluT3–/5‐HT– population overlapped with the former, whereas purely 5‐HT+ cells resembled the latter subgroup. Notably, we have not encountered fast spiking, rhythmic bursting 5‐HT+ neurones (Kocsis *et al*. 2006), possibly as a result of the different areas sampled in the present study. However, the electrophysiological phenotype of 5‐HT+ neurones reported in the present study is in agreement with the findings of previous studies (Mosko & Jacobs, 1974; Trulson & Jacobs, 1979; Urbain *et al*. 2006). The high firing rate of 5‐HT– cells may be the result of their more depolarized membrane potential and smaller activation gap, rendering this population highly responsive even to a small fluctuation of inputs. By contrast, 5‐HT+ neurones may be relatively resistant to changes of input patterns because of a hyperpolarized membrane potential, higher firing threshold (Beck *et al*. 2004) and various conductances favouring stereotypical discharge patterns (Tuckwell & Penington, 2014).

A key firing pattern feature is the rhythmic modulation characterizing neurone populations that are involved in the generation of oscillatory population patterns. Besides the well‐documented, regular discharge of 5‐HT+ neurones, rhythmically modulated firing was observed only in the case of one 5‐HT– neurone. The small depth and low stability of modulation implies non‐cell‐autonomous processes possibly generated by a combination of top‐down cortical influences and a fluctuating local activity. Indeed, low frequency LFP oscillations and the slow rhythmic modulation of discharge have been documented in the midbrain raphe nuclei (Hajos *et al*. 2007).

### Preference of forebrain activity states

All 5‐HT– neurones (both VGluT3+ and VGluT3–) fired faster during spontaneous theta compared to the non‐theta state. The MRR receives many afferents from brain areas involved in the regulation of theta activity; for example, cholinergic inputs from the pontomesencephalic tegmentum (Cornwall *et al*. 1990), GABAergic projections from theta‐related brainstem nuclei (e.g. nucleus incertus; Ma & Gundlach, 2015) or from the hypothalamus (Gervasoni *et al*. 2000), excitatory input from the lateral habenula (Wang & Aghajanian, 1977) and afferents from the medial septum (Fuhrmann *et al*. 2015). Any or the combination of these can feed theta‐coupled drive to the MRR. Additionally, cortical afferents from prefrontal areas are well known modulators of the midbrain raphe circuitry and may transmit fluctuating excitation (Hajos *et al*. 1998). Prefrontal or habenular stimulation was demonstrated to excite fast firing cells (previously assumed to be GABAergic) and inhibit the majority of tested 5‐HT+ neurones (Varga *et al*. 2001, 2003). In light of our results, at least a portion of the facilitated, putative GABAergic neurones would have belonged to the purely VGluT3+ subgroup identified in the present study. It can also explain some excitatory effect on 5‐HT+ neurones of prefrontal cortex stimulation: the latency and jitter conforms to either a slow‐conducting, low precision direct pathway or a disynaptic connection, in which prefrontal cortex input excites 5‐HT+ cells via VGluT3+ neurones. By contrast to the latter population, 5‐HT+ neurones (both VGluT3+ and VGluT3–) proved to be heterogeneous in terms of state‐preference. Some of them fired at higher frequency during non‐theta, slow oscillation‐dominated states, whereas others, such as 5‐HT– cells, emitted more action potentials when the hippocampus was governed by theta activity. The observation of heterogeneous state‐preference by 5‐HT+ populations recapitulates several earlier reports about variable relationship of slow firing, putative 5‐HT‐containing neurones to stages of the sleep–wake cycle or to activated/deactivated states of urethane‐anaesthesia (Viana Di Prisco *et al*. 2002; Urbain *et al*. 2006). Taken together, a higher impulse flow in 5‐HT– MRR pathways is associated with theta states, as opposed to a more uniform, state independent output of the 5‐HT+ populations.

### Effect of sensory stimulation

As the most important distinguishing feature, the four neurochemically separated MRR populations responded in a significantly divergent manner to tail pinch, a noxious sensory stimulus known to elicit an aroused brain state in urethane‐anaesthetized animals (Kramis *et al*. 1975). The effect of sensory stimulation on VGluT3+/5‐HT– neurones recapitulated the change coupled to the non‐theta to theta transition (i.e. persistent increase of activity for the whole duration of stimulation, except in case of one cell that stopped firing during tail pinch but was activated during spontaneous theta). Activating inputs can originate from several areas known to be activated by sensory inputs, such as pontomesencephalic cholinergic cell groups (Boucetta *et al*. 2014) or the medial septum (Swanson & Cowan, 1979), although VGluT3‐specific tracing is necessary to proceed beyond speculation. By contrast to the sustained response of VGluT3+/5‐HT– cells, a transient, short lasting acceleration was observed in the case of the 5‐HT+ populations (both VGluT3+ and VGluT3–). Sensory stimulus‐locked spiking of putative 5‐HT+ midbrain raphe neurones has recently been reported in freely‐moving mice exposed to a startle reflex‐inducing auditory stimulus (Ranade & Mainen, 2009), in agreement with earlier studies about sensory‐evoked responses in the raphe (Levine & Jacobs, 1992). Similar to the purely 5‐HT+ subgroup, VGluT3+/5‐HT+ neurones emitted spikes at stimulus onset but, resembling VGluT3+/5‐HT– cells, their activity remained elevated until termination of stimulation. This double phenotype may point to shared inputs with both pure populations (i.e. with VGluT3+/5‐HT– and VGluT3–/5‐HT+ cells). Alternatively, both of these populations may directly affect the dual identity cells. Interestingly, the VGluT3–/5‐HT–, putative GABAergic subgroup was the least affected by the delivery of a sensory stimulus. Our observations also raise the intriguing possibility that the fast spiking, theta coupled and sensory responsive neurones in previous studies may have belonged either to the ‘atypical’ 5‐HT+ or to the VGluT3+ subgroups, instead of being GABAergic.

### Modulation by forebrain oscillations

Phase coupling to forebrain oscillations did not differentiate the identified populations. Weak phase coupling either to prefrontal/hippocampal slow or hippocampal theta oscillation was detected in every neurochemical subgroup. A high prevalence of weak phase synchrony irrespective of neurochemical identity suggests that the MRR network may be constantly influenced by a complex and dynamically interfering mosaic of oscillating inputs (e.g. from prefrontal cortex, habenular complex or medial septum). However, our results also suggest that, instead of pacing any of the forebrain rhythms, the neurones that we recorded modulated them.

### Functional implications

Our data highlight the various facets of modulation implemented by MRR VGluT3+ neurones. A high firing rate and irregular spiking with occasional phase coupling to oscillations of VGluT3+/5‐HT– neurones are optimal for reliably conveying minute changes of input patterns, whereas a robust response to sensory stimuli is ideal for the reconfiguration of networks via highly target specific, temporally precise synaptic connections (Varga *et al*. 2009). Additionally, through extensive local axon arbours (Amilhon *et al*. 2010, Calizo *et al*. 2011)), VGluT3+ neurones may provide excitatory drive to 5‐HT+ cells, as suggested by Soiza‐Reilly & Commons (2011). Notably, MRR 5‐HT– neurones were shown to be unresponsive to 5‐HT1AR‐activation; thus, they can be decoupled from the activity of the serotonergic network (Beck *et al*. 2004). As such, the two 5‐HT– fast spiker populations (i.e. the glutamatergic and the GABAergic) may implement complementary feed‐forward regulation of the ascending 5‐HT+ system as a function of their extra‐raphe afferents. The shift of this excitation–inhibition balance would have a profound effect on the serotonergic modulation of forebrain operation. By contrast, the state‐dependent, stereotypical firing pattern of 5‐HT+ cells (both VGluT3+ and VGluT3–) may serve the regulation of vigilance states, which matches the classic view of the function of the serotonergic system (Jacobs & Azmitia, 1992). The stimulus‐locked activity of these cells, as suggested by recent studies, would signal the detection of salient stimuli (Ranade & Mainen *et al*. 2009), as well as switch the activity of target networks (Jackson *et al*. 2008). Additionally, the heightened discharge rate of VGluT3+/5‐HT+ cells would contribute to the stabilization of the post‐stimulation state.

In summary, the results of the present study have unravelled how the major MRR neurone classes share the regulation of spontaneously alternating or sensory‐evoked forebrain activity states. Any perturbation that alters the division of labour among the various components of the MRR network may have a disruptive impact on the operation of forebrain circuitry. We also raise the striking possibility that several pathological states previously connected to serotonergic dysfunction may actually be caused by impairment of the ascending glutamatergic system of the raphe.

## Additional information

### Competing interests

The authors declare that they have no competing interests.

### Author contributions

All of the experimental work and analyses were performed in the Laboratory of Cerebral Cortex Research, Institute of Experimental Medicine, Hungarian Academy of Sciences, Budapest, Hungary. AD helped design the study, performed experiments, analysed data and co‐wrote the manuscript. LNL, TL, CC, ZB and EP performed experiments and helped prepare the manuscript. GN contributed to analysis and interpretation of the data and helped prepare the manuscript. TFF contributed to the interpretation of the data and critically revised the manuscript. VV designed the study, performed experiments, helped in the analysis and co‐wrote the manuscript. All authors have approved the final version of the manuscript and agree to be accountable for all aspects of the work. All persons designated as authors qualify for authorship, and all those who qualify for authorship are listed.

### Funding

This work was funded by European Research Council ERC‐2011‐ADG‐294313‐SERRACO grant to TFF; Kerpel‐Fronius Young Investigators’ Grant to AD; János Bolyai Research Scholarship (BO/431/12/5) to GN; National Research, Development and Innovation Office (former Hungarian Scientific Research Fund) grant K 109790 to VV; and the National Brain Program of Hungary.
